# The Imbalance of Astrocytic Mitochondrial Dynamics Following Blast-Induced Traumatic Brain Injury

**DOI:** 10.3390/biomedicines11020329

**Published:** 2023-01-24

**Authors:** Fernanda Guilhaume-Correa, Alicia M. Pickrell, Pamela J. VandeVord

**Affiliations:** 1 Translational Biology, Medicine, and Health Graduate Program, Virginia Polytechnic Institute and State University, Roanoke, VA 24016, USA; 2 School of Neuroscience, Virginia Polytechnic Institute and State University, Blacksburg, VA 24061, USA; 3 Biomedical Engineering and Mechanics, Virginia Polytechnic Institute and State University, Blacksburg, VA 24061, USA; 4 Salem VA Medical Center, Salem, VA 24153, USA

**Keywords:** blast-induced traumatic brain injury, mild, acute, sub-acute, astrocytes, mitochondrial dynamics, fission and dynamin-related protein

## Abstract

Mild blast-induced traumatic brain injury (bTBI) is a modality of injury that has been of major concern considering a large number of military personnel exposed to explosive blast waves. bTBI results from the propagation of high-pressure static blast forces and their subsequent energy transmission within brain tissue. Exposure to this overpressure energy causes a diffuse injury that leads to acute cell damage and, if chronic, leads to detrimental long-term cognitive deficits. The literature presents a neuro-centric approach to the role of mitochondria dynamics dysfunction in bTBI, and changes in astrocyte-specific mitochondrial dynamics have not been characterized. The balance between fission and fusion events is known as mitochondrial dynamics. As a result of fission and fusion, the mitochondrial structure is constantly altering its shape to respond to physiological stimuli or stress, which in turn affects mitochondrial function. Astrocytic mitochondria are recognized to play an essential role in overall brain metabolism, synaptic transmission, and neuron protection. Mitochondria are vulnerable to injury insults, leading to the increase in mitochondrial fission, a mechanism controlled by the GTPase dynamin-related protein (Drp1) and the phosphorylation of Drp1 at serine 616 (p-Drp1^s616^). This site is critical to mediate the Drp1 translocation to mitochondria to promote fission events and consequently leads to fragmentation. An increase in mitochondrial fragmentation could have negative consequences, such as promoting an excessive generation of reactive oxygen species or triggering cytochrome c release. The aim of the present study was to characterize the unique pattern of astrocytic mitochondrial dynamics by exploring the role of DRP1 with a combination of in vitro and in vivo bTBI models. Differential remodeling of the astrocytic mitochondrial network was observed, corresponding with increases in p-Drp1^S616^ four hours and seven days post-injury. Further, results showed a time-dependent reactive astrocyte phenotype transition in the rat hippocampus. This discovery can lead to innovative therapeutics targets to help prevent the secondary injury cascade after blast injury that involves mitochondria dysfunction.

## 1. Introduction

Traumatic brain injury (TBI) is one of the most common injuries among the military and veteran population, with 82.3% being categorized as mild [1]. Mild blast-traumatic brain injury (bTBI) is a prevalent head injury among military combat personnel and veterans due to their exposure to explosives during armed conflict and training. TBI is a complex and heterogeneous injury, defined by sufficient external mechanical forces acting on the head and brain parenchyma, triggering a secondary insult of cellular and molecular dysfunction leading to a progressive change in the typical architecture and function of the brain. Clinical manifestations of mild bTBI include ongoing persistent cognitive deficits, including memory loss, emotional disorders, and anxiety problems [2,3,4,5,6,7]. When it comes to long-term memory, the hippocampus plays a crucial role, and preclinical models have shown neuronal death and astrocyte reactivity from mild bTBI in the hippocampus [8,9,10,11,12,13]. Treatments for bTBI remain elusive and are limited to symptom management because the pathophysiology of symptoms is poorly understood. In addition, there is a critical knowledge gap in understanding the cellular and molecular response post-blast exposure.

The morphologic and biological alterations that astrocytes experience in response to injury is known as astrocyte reactivity. The astrocyte reactivity phenotype in clinical and preclinical TBI pathology is primarily characterized by an increase in cell numbers, hypertrophy of their main cellular processes and soma, and alterations in the expression of the intermediate filament protein, glial fibrillary acidic protein (GFAP) [14,15,16,17,18]. Both in vitro and in vivo models of diffuse TBI and bTBI show that transition between astrocyte reactive phenotypes is highly dependent on injury mechanism and severity [13,19,20,21,22]. In the case of brain injury, the initial reactive phenotype is subtly controlled by the primary insult’s (mechanical) severity and followed by a secondary insult triggering a wide array of context-dependent intercellular and intracellular signaling mechanisms. Therefore, astrocyte reactivity is not a single stereotypical response [17,23,24,25]. Thus, all of these factors control the astrocytic response post-injury as either a positive, protective response, or a more harmful and detrimental response [26,27,28,29]. Many of those mechanisms by which astrocytes maintain and protect neurons are mitochondrial-associated mechanisms, including calcium homeostasis, buffering of excessive reactive oxygen species (ROS), regulating glutamate uptake and neuronal synaptic transmission [30,31,32,33,34]. Mechanical pressures from the original injury and downstream dysregulation from atypical metabolic cascades during the secondary insult can contribute to changes in mitochondrial dynamics after TBI [35]. Recent studies highlight the importance of proper astrocytic mitochondrial dynamics in a healthy brain and how altered mitochondrial dynamics could lead to both detrimental and protective astrocyte reactivity phenotypes in central nervous system (CNS) degenerative diseases and TBI pathologies [33,36,37,38,39,40]; however, the role of mitochondria in astrocytes during bTBI pathophysiology has been overlooked.

Mitochondria are important organelles. Besides being an essential source of adenosine triphosphate (ATP) generation, mitochondria regulate calcium levels and signaling, influence ROS levels and contain detoxifying antioxidants, control aspects of cell division and differentiation, and trigger intrinsic cell death pathways [41,42,43,44,45,46]. The mitochondrial structure is exceptionally dynamic and continually changing shape due to fission and fusion events, allowing it to respond to physiological cues or stress by modifying its structure, thus affecting function, since both are tightly associated. Mitochondria can display branching densely connected networks, a byproduct of fusion, or punctate and fragmented morphologies, a byproduct of fission. Therefore, the size and number of mitochondria, organellar quality control, and its transportation within the cell are all significantly influenced by fission and fusion events [41,44,47,48,49]. Recent studies conducted in vitro and in vivo suggested that astrocytic mitochondrial dysfunction, which favors fission over fusion post-TBI, is largely caused by dysregulation of the guanosine triphosphate (GTP)-binding dynamin-related protein (Drp1), a key player to carry out mitochondrial fission [38,50,51,52]. Drp1 is predominantly cytosolic and translocates to the outer mitochondrial membrane (OMM) during division [53,54,55,56,57]. Mitochondrial fission factor (Mff) and mitochondrial dynamics proteins of 49 and 51 kDa (MiD49 and MiD51) recruit Drp1 to sites where division will occur [58,59,60]. Post-translational phosphorylation regulates Drp1 recruitment at S616 or S637, either promoting or inhibiting its recruitment to the OMM, respectively [61,62,63]. Therefore, serine 616 or 637 phosphorylation affects mitochondrial morphology, and an increase in the astrocytic p-Drp1^s616^ protein levels were observed post TBI [38,50,51,52]. Changes in mitochondrial dynamics and their possible link to astrocyte reactivity have not yet been characterized concerning mild bTBI.

In this study, our aim was to provide evidence at the acute and sub-acute stages of the injury that astrocytic mitochondria dynamics in vitro and in vivo respond to a single mild bTBI. At these temporal time points, we found astrocytes altered their mitochondrial morphology by displaying fragmentation in response to bTBI. We attribute these increases in fission to Drp1 phosphorylation and further characterized the reactive phenotype response during these time points. The current study may point to a possible therapeutic window post-mild bTBI, leading to innovative therapeutic targets to help prevent secondary injury cascades involving alterations to astrocytic mitochondria.

## 2. Materials and Methods

The described study was carried out in accordance with protocols approved by the Virginia Tech Institutional Animal Care and Use Committee.

### 2.1. Primary Astrocytes Cell Culture

Brain cortices were isolated from two days post-natal (P2) Sprague-Dawley rats (Envigo, Dublin, VA, USA) by enzymatic digestion with 0.05% trypsin-EDTA for approximately ten minutes. Seven days after isolation, astrocytes were mechanically purified by gently shaking for 24-48 h. Astrocytes were maintained in Dulbecco’s modified Eagle’s medium (Gibco DMEM/F12; Cat#: 11320; ThermoFisher, Waltham, MA, USA) with 10% fetal bovine serum (Cat#: F2242; MilliporeSigma, Burlington, MA, USA) and 1% antibiotic-antimycotic (Cat#: 1540062; ThermoFisher, Waltham, MA, USA). Astrocyte cultures were stained with anti-GFAP (Cat#: ab7260; Abcam, Waltham, MA, USA) to ensure astrocyte purity and a homogenous astrocyte population (Figure S1). Astrocytes were seeded at a density of 1 × 105 cells per well and cultured for six days before mechanical exposure and were assigned to two groups according to data collection: Group I) primary astrocytes (PA) used for protein quantification were seeded in a standard six-well plate (Cat#: CLS3506; Corning, Somerville, MA), and Group II) PA cultures used for immunocytochemistry analysis were seeded in a treated 25mm round coverslip (Cat#: 229174; CellTreat, Pepperell, MA ) added to a six-well plate with three PA cultures per group.

### 2.2. High-Rate Overpressure Simulator: In Vitro Mechanical Exposure

**Figure 1:**
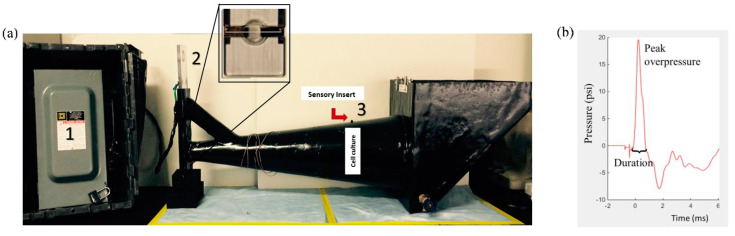
High-rate overpressure simulator (HOS)—in vitro mechanical injury model of bTBI. (**a**) The HOS is a water-filled chamber that exposes in vitro samples to high-rate overpressure via an exploding bridge wire mechanism. (1) Driver for the energy required (high electrical current); (2) Bridge wire mechanism (wire holder) and (3) cell culture plate location and sensory insert. Upon wire vaporization, the wave front travels down the test section of the cell culture plate denoted by “Cell culture”. (**b**) Pressure profile: The high-rate compression wave, as represented in the pressure profile, is meant to mimic an intracranial high-rate overpressure.

A high-rate overpressure simulator (HOS) (Figure 1a) was designed [64] based on studies conducted using intracranial pressure data from an in vivo blast TBI model using rodents [65]. The HOS is a one-chamber fluid-filled device that works through an exploding bridge wire mechanism by using a source of high electrical current in a closed electrical circuit containing a thin wire tightly suspended in two angled plates. This part of the circuit is submerged at the simulator’s small end. A capacitor and a voltage source act as the driver for the energy required, and upon discharge, a high current flow through the circuit up to the bridge wire leading to its vaporization, creating a high-rate compression wave that propagates through the conical section of the device exposing the cell culture plate. Peak overpressure data was recorded from a piezoelectric transducer (Cat# 8350C; Meggitt, Londonderry, NH, USA) sensor located in the device’s wall directly above and adjacent to the cell culture. The mechanical insult was performed, as described in Hlavac et al. [21]. Briefly, astrocyte cultures were exposed to a single isolated overpressure with an average of 20 psi (138 kPa) and approximately one-millisecond positive phase duration (Figure 1b). These metrics have been correlated to mild injury outcomes seen in blast rodent models [13,66,67]. Sham groups underwent the same steps without exposure to overpressure. Data analyses were performed at 4 h, 24 h, and 3 days post mechanical exposure.

### 2.3. Animal Procedures

**Figure 2:**
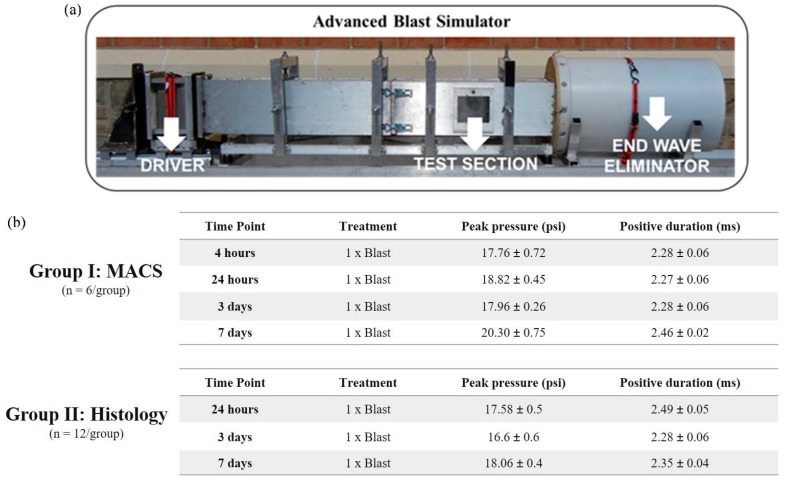
Advanced blast simulator (ABS)—in vivo primary blast wave injury model of bTBI. (**a**) (ABS consist of three distinct sections to create, develop, and dissipate the blast wave. Acetate membranes are ruptured following pressurization by helium gas located in the driver section. (**b**) Ten-week old Sprague Dawley rats (male, 250–300 g) were exposed to a single blast wave. The average peak pressure resulted in a blast wave magnitude of ~17–20psi, which induces a mild bTBI in rodents. (Group I) hippocampal astrocyte cell isolation by magnetic-activated cell sorting (MACS) technique for protein quantification and gene expression analysis. The group was divided into four-time points with a total of 6 animals per group (blast and sham): 4 h, 24 h, 3 days, and 7 days post single bTBI exposure; (Group II) brain tissue collection for microscopy analysis. The group was divided into three-time points with a total of 6 animals per group (blast and sham): 24 h, 3 days, and 7 days post bTBI exposure.

Adult male Sprague Dawley rats, aged 10 weeks and weighing approximately 250-300 g (Envigo, Dublin, VA, USA), were acclimated for several days before the blast experiment (12 h light/dark cycle) with ad libitum access to food and water. Prior to a single blast wave exposure, animals were anesthetized with 3% isoflurane and carefully placed in the advanced blast simulator (ABS). Animals were positioned in a prone position within a mesh sling designed to minimize flow hindrance and isolate primary blast injury with the elimination of acceleration/deceleration insult. Animals were then exposed to a single blast wave of approximately 17–20 psi, characterizing a mild bTBI. Sham animals received the same procedures except for the blast wave exposure. Animals were assigned to two groups according to data collection: Group I) hippocampal astrocyte isolation by magnetic-activated cell sorting (MACS) for protein quantification. The group was divided into four-time points with a total of six animals per group (blast and sham): four hours, 24 h, three days, and seven days post single bTBI exposure; Group II) brain tissue collection for microscopy analysis. The group was divided into three-time points with a total of 12 animals per group (blast and sham): 24 h, 3 days, and 7 days post single bTBI exposure (Figure 2a,b).

### 2.4. Advanced Blast Simulator: In Vivo Blast Wave Exposure

A custom advanced blast simulator (ABS) (200 cm × 30.48 cm × 30.48 cm) (Figure 2a) located at the Center for Injury Biomechanics (Virginia Tech University, Blacksburg, VA, USA) was used to generate a single blast wave. To produce, develop, and dissipate the blast wave, the ABS is divided into three parts. First, helium is compressed in the driver chamber using calibrated acetate membranes. Once passively ruptured, the blast wave is generated as it propagates through the transition chamber and reaches the animal in the “testing section” area. The blast wave continues to flow and will dissipate once reaching the end-wave eliminator located downstream of the testing chamber. As a final result, the animal was exposed to a single peak blast wave, recreating a free-field blast exposure. Pressure measurements were collected at 250 kHz using a Dash 8HF data acquisition system (Astro-Med, Inc, West Warwick, RI, USA). A customized Matlab (MathWorks, Inc, Natick, MA, USA) script was used to analyze pressure profiles and determine the impulse, duration, and rising time of the positive and negative phases. Rankine—Hugoniot relations and observed wave speed at the sensors within the ABS’s animal testing section were used to calculate the peak overpressure.

### 2.5. Magnetic-Activated Cell Sorting of Astrocytes

Hippocampal astrocytes were isolated by MACS protocol adapted from Holt and colleagues [68]. Following injury, animals were euthanized with exposure to carbon dioxide in an enclosed chamber. Following decapitation, brains were immediately removed, and the hippocampi was isolated. The isolated tissue was minced in the dissection media solution (Earl’s minimal essential media with 20mM glucose and 1Xantibiotic-antimycotic) and bubbled with gaseous carbogen (95% O_2_: 5% CO_2_) for up to five minutes. Papain, resuspension media, and albumin density gradients were prepared according to the manufacturing protocol (Table 1). The minced hippocampi were transferred to a 50 mL conical tube containing papain. The tissue–papain mixture was incubated for 20 min in a 37 °C water bath. After incubation, the tissue-papain mixture was titrated until homogenous and centrifuged. The resulting cell pellet was immediately suspended in the resuspension media. Next, the cell-resuspension mixture was carefully layered on the albumin density gradient solution and centrifuged.

The cell pellet was suspended in a buffer solution made of 0.5% BSA in PBS and filtered through a 70 mm BD Falcon cell strainer to remove any non-dissociated tissue. A hippocampal fraction containing all cells was first collected to be used as input control to determine hippocampal astrocyte purity test. Astrocytes were purified by the MACS technique [68] using superparamagnetic nanoparticles to tag the target cells retaining it in a column containing ferromagnetic steel wool placed in a magnetic separator. To increase the astrocytic fraction purity, microglia and oligodendrocytes precursor cells were removed first by incubation with MACS Cd11b+ microbeads and MACS myelin beads, respectively. The remaining cell mixture was incubated with Glt-1 antibody, a cell surface marker for astrocytes, followed by a secondary incubation with MACS anti-rabbit beads, allowing retention of astrocytes on a magnetic column (Table 1). The purified hippocampal astrocytes were immediately stored at −80 °C for later processing for protein quantification and gene expression analysis for purity (Figure S2).

### 2.6. Immunocytochemistry

PA cultures were washed three times with cold DPBS, fixed in warm (37 °C) 4% paraformaldehyde (pH 8) for 20 min, washed three times with DPBS at room temperature, and incubated in a solution of blocking buffer containing 10% normal goat serum (ThermoFisher; Cat#:50062Z) and 0.5% Triton-x 100 (Cat#: T9284, MilliporeSigma, Burlington, MA, USA) for 45 min at room temperature. Fixed cells were incubated, for three hours at 4℃, in blocking buffer with primary antibody TOMM20 at 1:100 dilution (Cat#: NBP1-81556; Novus Biologicals, Englewood, CO, USA) and GFAP at 1:100 dilution (Cat#: 13-0300; ThermoFisher, Waltham, MA, USA), this step was carefully conducted by placing the coverslip with upturned cells into a humidified chamber. Cells were washed four times with DPBS to remove unbounded primary antibody and then incubated for one hour at room temperature in blocking buffer with secondary antibody AlexaFluor 555 donkey anti-rabbit at 1:500 dilution and AlexaFluor 488 goat anti-mouse at 1:500 dilution. Post incubation, cells were gently washed three times with DPBS and incubated for ten minutes with DAPI in DPBS (1ug/mL) to visualize nuclei, followed by one wash with DPBS and one wash with ultra-pure water. Finally, the coverslips were mounted with Slow Fade-antifade reagent (Cat#: S36963; Invitrogen, Waltham, MA, USA) with downturned cells onto a tissue slide. Carbonyl cyanide 3-chlorophenylhydrazone (Cat#: ab141229; Abcam, Waltham, MA, USA) was used as a positive control. PA cultures that did not undergo blast or sham treatment were treated with 10µM CCCP for ten minutes prior to fixation. 

### 2.7. Immunohistochemistry

Rats were anesthetized with 5% isoflurane and perfused transcardially with saline followed by 4% paraformaldehyde (pH 7.5) at either 24 h, 3 days, and 7 days post sham or blast exposure. Post perfusion, brains were extracted and stored for 24 h at 4 °C in 4% paraformaldehyde solution to ensure proper brain fixation. Following the post-fixation period, brains were placed in 30% sucrose in PBS and embedded in optimal cutting temperature (OCT) medium (Sakura Finetek, Inc., Torrance, CA, USA) and frozen at −80 °C for cryostat sectioning. Coronal sections (40 μm) were prepared in a cryostat microtome (Thermo Scientific, Waltham, MA, USA) and stored in phosphate-buffered saline (PBS) with sodium azide at 4 °C prior to staining. Two random coronal sections (~Bregma −4.16 mm) were chosen per animal for immunohistochemistry analysis. For the immunohistochemistry procedure, all tissue samples were rinsed three times for five minutes with PBS and were incubated in a blocking buffer solution containing 10% normal goat serum (NGS) (ThermoFisher; Cat#:50062Z; ThermoFisher, Waltham, MA, USA) and 0.5% Triton-x 100 (Cat#: T9284; MilliporeSigma, Burlington, MA, USA) for 1 h at room temperature. Tissue samples were incubated for 16–18 h at 4 °C in blocking buffer with primary antibody GFAP at 1:1000 dilution (Cat#: 13-0300; Invitrogen, Waltham, MA, USA). The following day, tissue samples were washed three times for five minutes in PBS and incubated for 1.5 h at room temperature in a blocking buffer with secondary antibody AlexaFluor 488 goat anti-mouse at 1:1000 dilution. Following the incubation, tissue samples were washed three times for five minutes with PBS and incubated for 10 min with DAPI in PBS (1 ug/mL) to visualize nuclei, followed by one wash with PBS. Finally, tissue samples were mounted and coverslipped with Slow Fade-antifade reagent (Cat#: S36963; Invitrogen, Waltham, MA, USA). 

### 2.8. Mitochondrial Morphology Analysis

Fluorescence images were acquired using a confocal microscope (Zeiss, Jena, Germany) with a 63x/1.40 oil objective lens, and the Z-stack series consisted of 0.25 µm slice intervals with 12 slices per sample. Images were stacked into a single 2D image using the Zeiss Zen blue 2 software processing tool called “extended depth of focus.” A total of four images per PA culture were acquired with approximately three cells per image area in three biological replicates; therefore, a total of ~36 cells per group (sham, blast, and CCCP) were analyzed. Additional measurements of mitochondrial morphological characteristics were made using Fiji (ImageJ, USA) [69]. An image macro toolset called Mitochondrial Network Analysis (MiNA) software uses existing ImageJ plugins to help with a semi-automated analysis of mitochondrial morphology in cultured cells [40,70,71]. In addition, MiNA software presents optional pre-processing settings to help to enhance image quality prior to analyses. For analysis, MiNA starts by binarizing and then skeletonizing the original image to obtain a morphology skeleton to calculate parameters to quantitatively capture the mitochondrial morphology (Figure S3). Finally, MiNA finishes with a summary of data parameters that classify the mitochondrial morphology as individuals, networks, and mitochondrial footprint. Individual mitochondria (rods, punctate, and large/round shapes) and all other objects in the image without a junction pixel are recognized by the MiNA software. All items in the image that have at least one junction are categorized as networks.

### 2.9. Fluorescence Microscopy Analysis for GFAP Marker

All tissue samples were imaged using Fluorescence microscope (Axio Observer 7; Zeiss, Dublin, California) at a 20X objective lens to analyze the astrocyte reactivity phenotypes. Images of hippocampal sections containing all four sub-regions: dentate gyrus (DG) and CA1, 2, and 3 were acquired for GFAP analysis. All images were processed and quantified using Fiji (ImageJ, USA) software. First, the background was subtracted using the built-in function on the software. Then, a threshold value was determined and applied equally to all images to isolate GFAP expression from any background fluorescence. After preparing all images, ImageJ software quantified GFAP fluorescence by three specific parameters:

1. Integrated density of fluorescence: Measures the GFAP positive fluorescence signal level using gray pixel intensity.

2. Area fraction: Quantifies the percentage of GFAP positive signal within the region of interest.

3. Mean area per cell: It is acquired by the “analyze particles” function with the pixel area size threshold of 0.004, excluding slight pixel noise and extracting objects of interest.

Data were acquired from the average of the replicate images, 2 coronal sections per animal with 4 images per subregion. The final data were determined per animal for each hippocampal sub-region and a total hippocampal expression by adding the data averages from each subregion.

### 2.10. Protein Extraction

Phase separation with chloroform was followed by Trizol (Cat# 15596018; ThermoFisher, Waltham, MA, USA) treatment on the samples. After extracting RNA and DNA, proteins were recovered from the Trizol phenol phase. The first step in protein precipitation was isopropanol treatment, followed by three 20-min washes with a 0.3 M guanidine hydrochloride in 95% ethanol solution and one wash with a 20-min 100% ethanol solution. Next, the protein was re-suspended in a 1:1 solution of 1% sodium dodecyl sulfate solution and 8M urea in 1M Tris-HCl (pH 8). The protein was then further homogenized by sonication for an additional ten seconds, followed by ten minutes of incubation at 55 °C. BCA assay (Pierce; Cat#: 23225; ThermoFisher, Waltham, MA, USA) was used to measure the total protein in the samples in preparation for Western blotting analysis.

### 2.11. Western Blotting

Protein samples were produced per the manufacturer’s instructions, and a capillary-based automatic Western system (Wes Protein Simple; Bio-techne, Minneapolis, MN, USA) was used for the protein quantification of each studied marker. Wes supplies purchased from Protein Simple were separation modules (Cat#: SW-004; Bio-techne, Minneapolis, MN, USA), anti-rabbit (Cat#: DM-002; Bio-techne, Minneapolis, MN, USA), and anti-mouse (Cat#: DM-001; Bio-techne, Minneapolis, MN, USA) detection modules. Primary antibodies used to probe total protein samples were GFAP (Cat#: ab7260; dilution 1:100; Abcam, Waltham, MA, USA), Drp1 (Cat#: NB110-55288; dilution 1:500; Novus Biologicals, Englewood, CO, USA), p-Drp1^s616^ (Cat#: 4494S; dilution 1:20; Cell Signaling, Danvers, MA, USA), and p-Drp1^s637^ (Cat#: 4867S; dilution 1:20; Cell Signaling, Danvers, MA, USA). Protein levels were calculated using Compass for SW software v.6.1 (Protein Simple; Bio-techne, Minneapolis, MN, USA) from area measurements obtained from electropherograms at the same exposure period for all data plates. The Wes software determines target protein levels by computing the area under the curve using uniform peak fits across all data. Therefore, the Wes bands shown in the data are digitized images meant to be a qualitative visual comparison of how the target protein traveled through the Wes capillary-based automatic system. According to the target protein, samples were loaded with the same protein concentration (mg/mL) in each well and normalized to that total protein.

### 2.12. Statistical Analysis

Statistical comparisons were conducted between groups using GraphPad Prism version 9 for Windows (GraphPad Software, San Diego, CA, USA). A two-way ANOVA was conducted to analyze overall significant differences amongst groups, followed by a Tukey’s honestly significant difference (Tukey’s HSD) test to analyze which specific group’s average differed. For group comparisons within a single individual, a Student’s *t*-test was used. Levene’s tests were employed to evaluate the equality of variances, and the Kolmogorov-Smirnov test was used to check the assumptions for normality. A logarithmic adjustment was carried out to undertake statistical comparisons in the case that the data were not normal. A *p*-value of 0.05 or less was regarded as statistically significant, and residual analysis was used to identify statistical outliers. The letter “n” stands for the total number of samples per group. All data have been represented as the mean ± standard error of the mean (S.E.M.). All data have been normalized to their respective shams for each time point.

## 3. Results

### 3.1. High-Rate Mechanical Insult Induces Acute Astrocytic Mitochondrial Fragmentation

**Figure 3:**
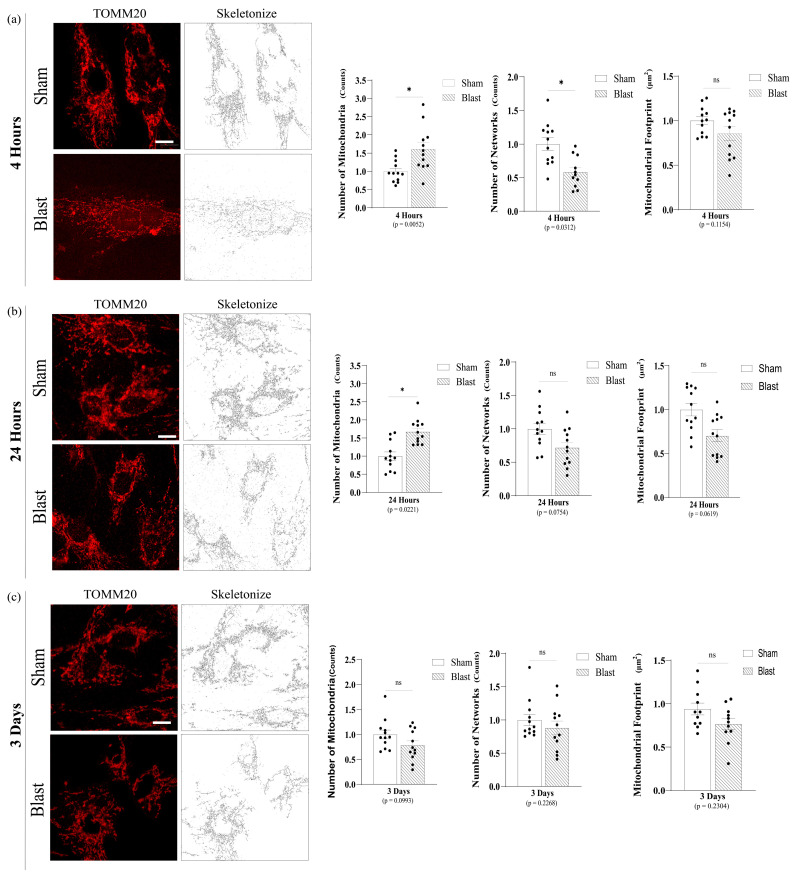
Mitochondrial Network Analysis (MiNA) software descriptors from astrocyte mitochondrial morphology post single mechanical exposure—in vitro model of bTBI. (**a**) Representative images (63x) of TOMM20 and skeletonized for data acquisition at 4 hours post single mechanical exposure. Astrocytes presented a significant increase in the number of individuals fragmented mitochondria (puncta and rod) and a small number of networks. (**b**) Representative images of TOMM20 and skeletonized for data acquisition at 24 h post single mechanical exposure. Astrocytes still presented a significant increase in the number of individuals fragmented mitochondria (puncta and rod), displayed lower number of networks and mitochondrial footprint (the total area in the cell expressing mitochondrial marker TOMM20), although not statistically significant. (**c**) Representative images of TOMM20 and skeletonized for data acquisition at 3 days post single mechanical exposure, and there is no significant difference in the number of individuals, networks, and mitochondrial footprint between sham and overpressure groups. (Each point represents average of 3 cells per image with a total of 4 images per group; data are mean ± SEM; * *p*-value represents ≤ 0.05; ns-value represents not significant; Scale bar = 10 μm).

To analyze mitochondrial morphological features such as fragmented mitochondria and number of networks, astrocytic mitochondria were labeled by immunocytochemistry for the outer mitochondrial membrane protein TOMM20. The mitochondrial network was examined using MiNA software, whereas fragmented mitochondria consist of any rods, punctate, and large/round shape in morphology. Mechanical blast insult significantly increased the number of fragmented astrocytic mitochondria (*p* = 0.0052) with a decrease in the number of mitochondrial networks (*p* = 0.0312) at 4 h (Figure 3a). Furthermore, at 24 h, the number of fragmented mitochondria was still significantly increased (*p* = 0.0221); however, the number of networks were no longer significantly changed (Figure 3b). 

Notably, significant differences between the blast group and sham were resolved in 3 days. Therefore, the astrocyte mitochondrial network presented an acute but transient fragmentation with a smaller number of networks post high-rate mechanical insult (Figure 3c). Positive control samples demonstrated that astrocytic mitochondria treated with the mitochondrial uncoupler CCCP were severely fragmented with significantly increased fragmented mitochondria (*p* < 0.0001) with a significantly reduced total mitochondrial footprint (*p* < 0.0001) (Figure S4) as expected, demonstrating that the MiNA software successfully identified and characterized morphological features of the astrocytic mitochondrial network.

**Figure 4:**
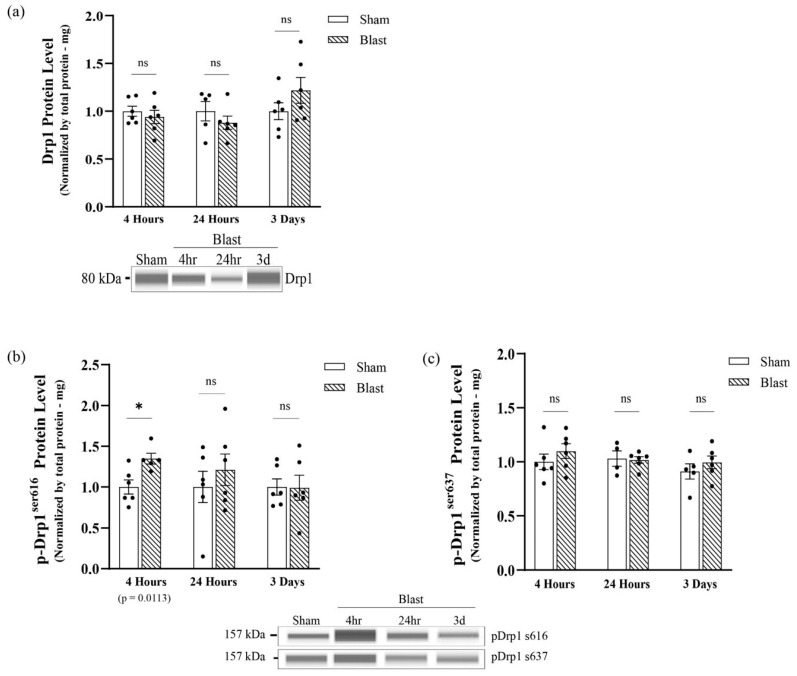
Analysis of total GTP-DRP1 protein levels and total DRP1 post-translational modification protein levels at serine 616 and serine 637 post single mechanical exposure—in vitro model of bTBI. Western blotting was conducted using an automatic capillary-based system (Wes, Protein Simple) to look at relative protein quantification to identify possible fission post-overpressure exposure. Only for qualitative visual comparison, digitized bands illustrate how proteins moved through the capillary system on the Wes. (**a**) There were no significant changes in DRP1 protein levels at 4 h, 24 h, and three days following the mechanical insult. (**b**) The total levels of p-DRP1^s616^ protein were increased by four hours and returned to physiological levels by three days post single mechanical exposure. (**c**) The total levels of p-DRP1^s637^ protein were maintained at physiological levels at all-time points post single mechanical exposure (*n* = 6/group; data are mean ± SEM; * *p*-value represents ≤ 0.05; ns-value represents not significant).

Due to an increase in fragmentation, we assessed if this mitochondrial phenotype was due to Drp1 recruitment dependent on phosphorylation, which coordinates mitochondrial fission dynamics. First, Western blotting analyses were performed to determine if exposure to the mechanical insult altered Drp1 protein levels at 4 hours, 24 h, and 3 days following the mechanical insult. Results indicated no significant changes in Drp1 protein levels between blast and sham groups at all-time points (Figure 4a). p-Drp1^s616^ triggers mitochondrial fission by promoting Drp1 translocation from the cytoplasm to the OMM, and on the other hand, p-Drp1^s637^ suppresses mitochondrial fission by inhibiting Drp1 translocation. The levels of p-Drp1^s616^ total protein were significantly increased (*p* = 0.0309) at 4 hours post mechanical insult (Figure 4b), indicating an increase in the activity of Drp1-mediate mitochondrial fission, and no significant changes were seen in total p-Drp1^s637^ at this time point (Figure 4c). Conversely, total p-Drp1^s616^ returned to sham levels at 24 h and 3 days post mechanical insult (Figure 4b). Furthermore, levels of p-Drp1^s637^ were maintained at sham levels at all-time points in the mechanical insult group (Figure 4c). This data correlated with the fragmented mitochondrial outcomes we observed in the primary astrocytes culture, further indicating that mitochondrial fission events increased at an acute point post mechanical insult and returned to physiological levels by 3 days.

**Figure 5:**
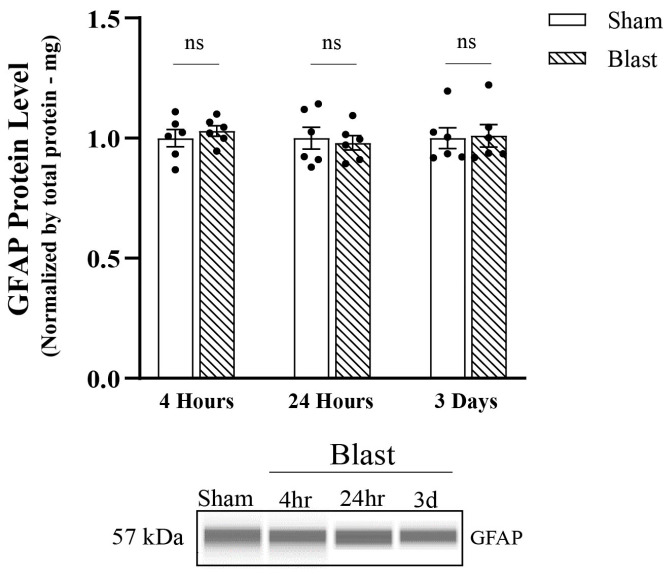
Analysis of total GFAP protein levels post single overpressure exposure—in vitro model of bTBI. Western blotting was conducted using an automatic capillary-based system (Wes, Protein Simple) to look at relative protein quantification to identify possible fission post-overpressure exposure. Only for qualitative visual comparison, digitized bands illustrate how proteins moved through the capillary system on the Wes. Total levels of GFAP protein were maintained at physiological levels at all-time points. (n = 6/group; data are mean ± SEM; ns-value represents not significant).

We next assessed the astrocyte reactivity by Western blotting. GFAP protein levels were quantified; however, no significant changes in the total protein levels of GFAP between blast and sham groups appeared between all-time points (Figure 5).

### 3.2. Single Blast-Induced TBI Exposure Increased Drp1^s616^ Phosphorylation In Vivo

**Figure 6:**
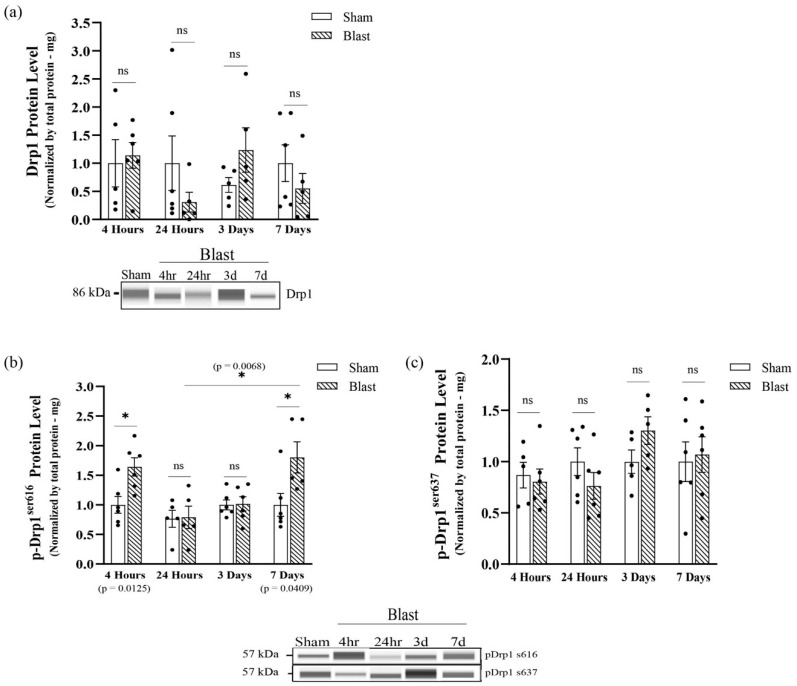
Analysis of total GTP-DRP1 protein levels and total DRP1 post-translational modification protein levels at serine 616 and serine 637 post single mechanical exposure—in vivo model of bTBI. Western blotting was conducted using an automatic capillary-based system (Wes, Protein Simple) to look at relative protein quantification to identify possible fission post-overpressure exposure. Only for qualitative visual comparison, digitized bands illustrate how proteins moved through the capillary system on the Wes. (**a**) There were no significant changes in DRP1 protein levels at 4 h, 24 h, 3 d, and 7 d following blast wave exposure. (**b**) The total levels of p-DRP1^s616^ protein were acutely increased by 4 h, returned to physiological levels at 24 h and 3 d and shifted back to an increase at 7 d post blast wave exposure. (**c**) The total levels of p-DRP1^s637^ protein were maintained at physiological levels at all-time points. (n = 6/group; data are mean ± SEM; *p-value represents ≤ 0.05; ns-value represents not significant).

To confirm our findings in an in vivo model of blast injury, we extracted protein from purified astrocytes derived from the hippocampi of rats at the acute time points of 4 hours and 24 h and at the sub-acute time points of 3 days and 7 days following blast wave exposure to determine if bTBI alters astrocytes Drp1 protein levels. Results indicated no significant changes in total Drp1 protein levels between groups at all-time points, which reflected results that we found in our astrocyte cultures (Figure 6a). The activation of Drp1 protein by p-Drp1^s616^ activates Drp1 translocation to the OMM. Our results indicated that, at 4 hours following blast wave exposure, a significant increase in p-Drp1^s616^ protein levels occurred (*p* = 0.0125). Furthermore, the total level of p-Drp1^s616^ protein was restored to sham levels at 24 h and 3 days, whereas at 7 days, levels of p-Drp1^s616^ protein were shifted back to a significant increase compared to the sham group (*p* = 0.0409) (Figure 6b). Furthermore, levels of p-Drp1^s637^ protein were not statistically significant across all time points (Figure 6c). p-Drp1^s616^ levels normalized to Drp1 from each individual animal reflected the same findings except that p-Drp1^s637^ levels were significantly higher at 3 days (*p* = 0.03444) (Figure S5).

These data indicated that changes in mitochondrial morphology in astrocytes are most likely due to the primary mechanical injury in blast, as reflected by the changes detected at 4 h but not at the sub-acute time points. However, p-Drp1^s616^ is significantly increased again at the 7 d time point, indicating that secondary insults may be responsible for more chronic changes to mitochondria post-blast.

### 3.3. Blast-Induced TBI Exposure Induces Hippocampal Astrocyte Reactivity

**Figure 7:**
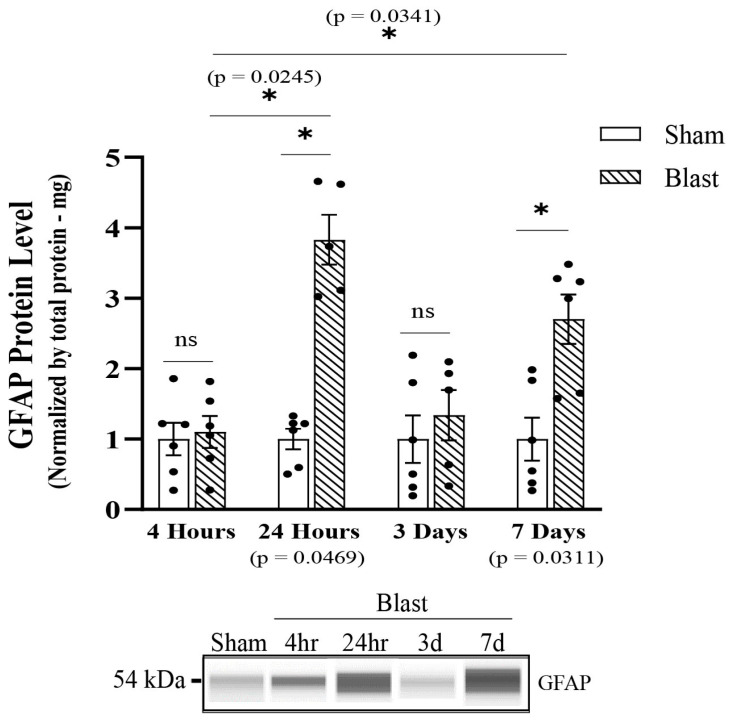
Analysis of total GFAP protein levels post single overpressure exposure—in vivo model of bTBI. Western blotting was conducted using an automatic capillary-based system (Wes, Protein Simple) to look at relative protein quantification to identify possible fission post-overpressure exposure. Only for qualitative visual comparison, digitized bands illustrate how proteins moved through the capillary system on the Wes. Total GFAP protein levels were increased at 24 h, returned to physiological levels by 3 d and raised backed up by 7 d post blast wave exposure. (n = 6/group; data are mean ± SEM; * *p*-value represents ≤ 0.05; ns-value represents not significant).

**Figure 8:**
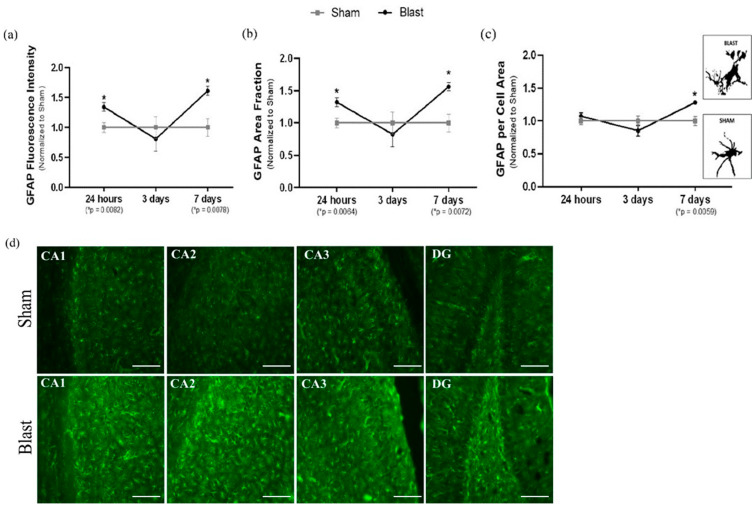
To provide a comprehensive analysis of astrocyte reactivity in the adult rodent hippocampus, fluorescence microscope was used to observe changes in GFAP. A total hippocampal expression was acquired by adding the data averages from each sub-region and three parameters were used by ImageJ Software in order to quantify astrocyte reactivity. (**a**) GFAP fluorescence intensity: there were a significantly elevated GFAP fluorescence intensity in the total hippocampus at 24 h and 7 days post blast wave exposure. The higher intensity of fluorescence may indicate astrocyte reactivity following bTBI. (**b**) GFAP area fraction: the area fraction that the GFAP signal occupied signifies the percent of positive signal within a given region, which was significantly elevated at 24 h and 7 days in the total hippocampus post blast wave; therefore, indicating an increased in the number of astrocytes to the total hippocampal injured region. (**c**) GFAP per cell area: there were a significantly increased GFAP mean area per cell at 7 days post blast wave, indicating a hypertrophy of the astrocytes body and main processes. Furthermore, a view of individual astrocytes (GFAP) in both sham and single blast animals showed different sizes of cell bodies (soma) and processes. (**d**) Representative images (20×) of the hippocampus sub-regions (CA1, CA2, CA3 and DG) seven days post blast wave. (Fluorescence Microscopy group: n = 12/group; data are mean ± SEM; * *p*-value represents ≤ 0.05; Scale bar = 57 μm).

Next, we focused on characterizing the time course of hippocampal astrocyte reactivity from adult male rats’ exposure to a blast wave. To assess the reactive phenotype, protein and fluorescence levels of GFAP were analyzed by Western blotting and microscopy at 4 h, 24 h, 3 days, and 7 days. Results indicated a significant increase in total GFAP protein levels starting at 24 h (*p* = 0.0469) (Figure 7a), which also corresponded with an increase in GFAP fluorescence intensity (*p* = 0.0082) (Figure 8a), as well as an increase in GFAP area fraction (*p* = 0.0064) post-single blast exposure (Figure 8a). Increased area fraction may indicate the increased number of astrocytes in the injured hippocampus. The response was shifted back to physiological (sham) levels at three days post-blast wave (Figure 7a and Figure 8a). At 7 days post blast wave, hippocampal astrocytes presented a significant increase in total GFAP protein (*p* = 0.0311), GFAP fluorescence intensity (*p* = 0.0078), GFAP area fraction (*p* = 0.0072) (Figure 8a), and a further increase in GFAP per cell area (*p* = 0.0059) (Figure 8a), and presumably, this could signify changes in the cytoskeleton and main processes. Furthermore, each hippocampal sub-region was analyzed separately with the same data parameters. The results indicated that the total hippocampus data correctly represented a diffuse injury across the hippocampus since a significant difference was observed in each sub-region at 24 h and seven days, and no changes were observed at 3 days post blast wave exposure (Figure S6). Together, those data indicate the astrocyte reactivity phenotype following mild bTBI upregulates GFAP and is a well-represented marker for reactivity in TBI.

In conclusion, both in vitro and in vivo blast TBI models indicate that astrocyte mitochondrial respond to a single mechanical and blast wave with an increase in Drp1 phosphorylation at S616. We further characterized an oscillatory astrocyte reactivity phenotype in vivo. Astrocyte reactivity was demarked by an increase in GFAP protein and an increase in the number of cells, which was later accompanied by the hypertrophy of soma body and processes. Furthermore, we demonstrated similar mitochondrial dynamics response patterns between the in vitro and in vivo bTBI models, indicating that our in vitro model provides a novel tool to study intracranial pressures effects on cultured cells.

## 4. Discussion

Astrocytes play an essential role in a healthy brain and proper maintenance of the CNS. However, there is increasing debate over the different functions associated with astrocyte reactivity and their response to the injured brain. They may play a dual role in TBI by contributing to the repair of damaged tissue and the return to homeostasis or contribute to deleterious effects by causing scar tissue formation and therefore inhibit neuronal axonal plasticity [18,29]. Mitochondria are essential for several astrocytic processes, including glutamate metabolism, calcium signaling, fatty acid metabolism, antioxidant generation, and inflammatory activation [31,38,52]. Studies emphasized the significance of astrocytic balanced mitochondrial dynamics (fission and fusion) in a healthy brain and the potential repercussions of altered mitochondrial dynamics from both protective and maladaptive astrocyte reactivity patterns in TBI detrimental outcomes [33,34,36,37,39,40]. As a result of their influence over such a wide range of secondary injury sequelae, astrocytic mitochondria have risen to prominence as a therapeutic target in neurotrauma research. The current study is the first to address a time-dependent response of astrocytic reactive phenotype and astrocytic mitochondrial dynamics by characterizing the GTP-protein Drp1 and its role in mitochondrial fission events using both in vitro and in vivo models of mild bTBI. 

bTBI combines the interplay between the primary mechanical insult and the subsequent secondary biochemical cascade. Initially starting as a mechanical injury, it is critical to comprehend the mechanical insult characteristics and forces that affect the brain, specifically how they are transferred to the mitochondria. Astrocytes are mechanically sensitive cells that respond to mechanical overpressure insult [21,72,73,74,75,76,77]. Results from this study indicated that the HOS induced differential remodeling of the astrocytic mitochondrial network, leading to an increase in fragmentation by presenting a higher number of individual mitochondria and a lower number of mitochondrial networks as early as four hours following insult. Drp1 post-translational modifications, such as the phosphorylation at the serine 616 site, significantly increased at four hours, indicating an increase in Drp1 recruitment and, consequently, increased fission events in the blast group in in vitro and in vivo bTBI models. In the in vivo bTBI model, this was later accompanied at 24 h post-blast by significantly increased GFAP protein levels and astrocytic cell numbers, indicating an initial astrocyte reactivity phenotype within the hippocampus. Considering these changes to mitochondrial fission occurred in the absence of other cells in our primary cultures at 4 h, our data suggest that the mechanical blast wave is a main contributor to these mitochondrial alterations. Increased p-DRP1^s616^ present at 7 days post injury in astrocytes in our preclinical model suggest that secondary effects from the blast from other cell types could be responsible for mitochondrial remodeling at later time points. However, more research will be required to understand how mitochondria recover from the initial blast after fragmentation between the 4- and 24-h time points and the pathophysiological outcomes to the initial fission events. 

GFAP is a critical intermediate filament protein responsible for astrocyte cytoskeleton structure playing a crucial role in controlling astrocyte motility by giving astrocytic processes structural stability [15]. The acute stages of astrocyte reactivity post-TBI could present reparative characteristics [23,25,29]. Mitochondrial fragmentation generates a localized redox signaling event at the site of the injury to aid in plasma membrane closure, and cells lacking Drp1 were deficient in this repair process [78]. An acute increase in mitochondrial fragmentation could be essential to facilitate the proper function of astrocytes since their microdomains are crucial to match the local calcium activity to facilitate calcium buffering [79]. Increasing neuronal activity causes transitory mitochondrial remodeling in astrocytes by encouraging the immobility of mitochondria in their tiny processes near high neuronal synaptic activity, allowing astrocytes to properly fit the local energy requirements and calcium buffering requirements [79,80,81,82]. 

Post mechanical insult, at 3 days, the results presented a decrease in fragmented mitochondria with mitochondrial network remodeling returning to physiological levels. This phenomenon could further indicate that mitochondrial fission serves as an early form of cellular protection by clearing damaged mitochondria before the start of apoptotic pathways [83]. Mitochondrial fission events can enable damaged mitochondria to dissociate from the network and subsequently allow the degradation of the damaged portion of the mitochondrial network by mitophagy, a selective form of autophagy to degrade damaged or superfluous mitochondria by the lysosome [73,83,84,85,86]. An in vitro study reported that astrocytes responded to inflammatory stimuli, a typical secondary insult post-TBI, with a prompt autophagic response, and blocking this mechanism led to a failure in regenerating mitochondrial integrity ultimately affecting astrocyte survival [50]. Furthermore, the remodeling of the astrocytic mitochondrial network correlated with decreased Drp1 since the observed p-Drp1^s616^ protein levels were restored to physiological levels by 3 days post mechanical insult, indicating a restoration of mitochondrial dynamics. Curiously, the same phenomenon was observed in the astrocyte reactivity phenotype, with GFAP being restored to physiological levels by 3 days post-blast wave exposure. However, this astrocytic homeostatic phenotype was again disrupted 7 days post-blast, correlating with an increase in p-Drp1^s616^ levels, while p-Drp1^s637^ levels were maintained at physiological levels. This was accompanied by GFAP shifting back to higher increase in total protein levels combined with the increase in astrocytic cell number and additional changes in the cytoskeleton, indicating a possible start of the reactive phenotype and, as a result, a possible detrimental bTBI outcome. 

Together, these current data are the first to demonstrate that a mechanical insult triggers an acute and sub-acute reactive astrocyte phenotype accompanied by alterations in astrocytic mitochondrial dynamics. It is noteworthy that those alterations were first seen in the mitochondrial structure as early as 4 hours post mild mechanical insult. An oscillatory reactive phenotype was observed by upregulation of GFAP, and it could be represented as an astrocytic protective response acutely after mild bTBI since a homeostasis stage was achieved at 3 days post-injury. However, it can lead to a phenotypic transition into a severe reactive state, whereas it can distinctly extend into a highly proliferative, scar-forming phenotype due to a chronic-state injury [28,87,88,89,90,91].Although GFAP is a well-represented marker for astrocyte reactivity in TBI [14,18,92,93], it would be important to further characterize the astrocyte reactive phenotype by looking at other markers, such as vimentin, nestin, complex C and aldolase C.

Taking this into account and knowing that mitochondrial dynamics respond to physiological and stress insults by remodeling and adapting its shape and structure, thereby altering its function, protecting mitochondria by maintaining its dynamic balance is critical. In conclusion, based on the data presented in two preclinical models of bTBI, mitochondrial fragmentation occurs acutely after bTBI and is necessary to promote astrocytic protection and cytoskeleton plasticity, allowing astrocytes to present a protective phenotype in critical phases of early secondary bTBI insult. If persisting, it could lead to a loop of detrimental intercellular signaling in the astrocytes, such as lack of proper calcium buffering, increase in the oxidative stress environment activating the NF-kB pathway, and resulting in the release of astrocytic pro-inflammatory mediators [32,50].

## Figures and Tables

**Table 1:** List of supplies used for magnetic-activated cell sorting technique.

Reagent	Vendor	Catalog Number
Carbogen (95% O_2_: 5% CO_2_)	AirGas (Christiansburg, VA, USA)	X02OX95C2003102 (CGA 296)
Papain dissociation system	Worthington (Lakewood, NJ, USA)	LK003150 (PDS)
Falcon cell strainers (70 mm)	Fisher Scientific (Waltham, MA, USA)	08-771-2
QuadroMACS	Miltenyi Biotec (Waltham, MA, USA)	130-091-051
LS columns MACS	Miltenyi Biotec (Waltham, MA, USA)	130-042-401
MACS Cd11b+ microbeads	Miltenyi Biotec (Waltham, MA, USA)	130-093-634
Myelin isolation microbeads	Miltenyi Biotec (Waltham, MA, USA)	130-104-257
Anti-rabbit IgG microbeads	Miltenyi Biotec (Waltham, MA, USA)	130-048-602
Anti-EAAT (GLT-1) antibody	Alomone Labs (Jerusalem, Israel)	AGC-022
Taqman GFAP qPCR primer	Thermo Fisher Scientific (Waltham, MA, USA)	Rn00566603_m1
Taqman MBP qPCR primer	Thermo Fisher Scientific (Waltham, MA, USA)	M01399619m1
Taqman Rbfox1 qPCR primer	Thermo Fisher Scientific (Waltham, MA, USA)	Rn01464214_m1
Taqman Itgam qPCR primer	Thermo Fisher Scientific (Waltham, MA, USA)	Rn00709342_m1

## Data Availability

All datasets presented in this study are included in the article main figures and Supplementary Materials.

## Supplementary Materials

The following supporting information can be downloaded at: https://www.mdpi.com/article/10.3390/biomedicines11020329/s1, Figure S1: Primary astrocytes cultures; Figure S2: Group II: Magnetic-activated cell sorting (MACS)—Hippocampal astrocyte cell; Figure S3: Mitochondrial Network Analysis (MiNA) software description and image acquisition; Figure S4: Mitochondrial Network Analysis (MiNA) software descriptors show differences in astrocyte mitochondrial morphology in Sham and CCCP positive control groups; Figure S5: Analysis of p-DRP1s616 and p-DRP1s637 protein ratio to total DRP1 post single; Figure S6: Sub-regions of the hippocampus were further analyzed for GFAP to identify if the total hippocampus data correctly represent a diffuse injury across the adult rodent hippocampus at 24 h, three days and seven days post single mild blast wave exposure mechanical exposure isolation.
