# Supplementary material for: The Imbalance of Astrocytic Mitochondrial Dynamics Following Blast-Induced Traumatic Brain Injury
Source: Biomedicines. 2023 Jan 24;11(2):329. doi: 10.3390/biomedicines11020329 (PMC9953570; doi:10.3390/biomedicines11020329)
Supplement: Supplementary file 1 [file biomedicines-11-00329-s001.zip › biomedicines-2045968-supplementary.pdf]

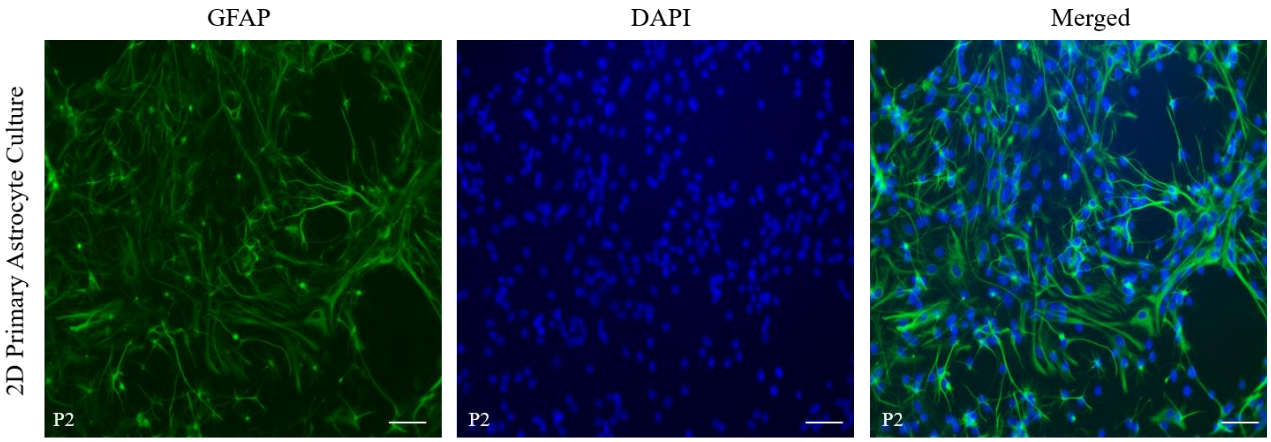

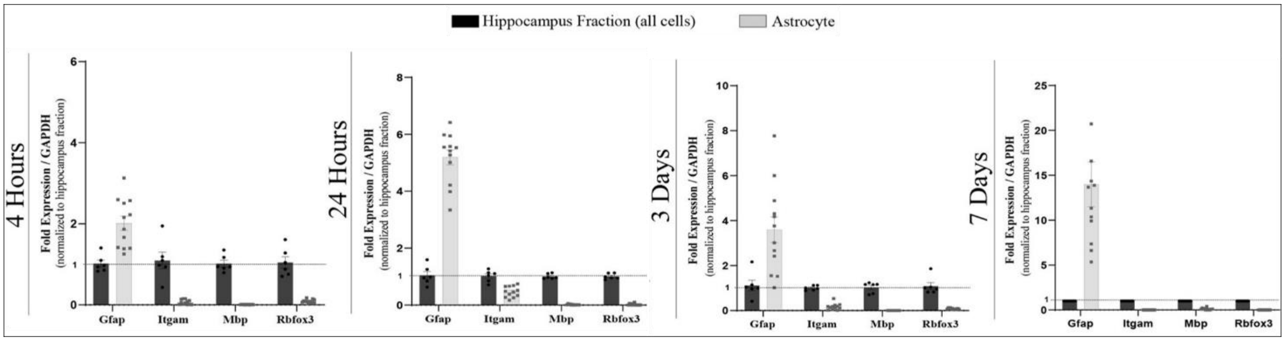

Supplementary Figure S1. Primary astrocytes cultures. Image of GFAP-positive primary astrocyte cell culture. Two days post-natal (P2) Sprague-Dawley primary astrocytes were stained with GFAP antibody to make sure the study population was purified (Scale bar = 75 µm).

Supplementary Figure S2. Group II: Magnetic-activated cell sorting (MACS) – Hippocampal astrocyte cell isolation. Taqman probes for GFAP (astrocytes), ITGAM (microglia), RBFOX3 (neurons) and MBP (oligodendrocytes) were used to measure gene expression of respective cell types with GAPDH used as a housekeeping gene. A hippocampus fraction with all cells present was collected to be used as an input control for purity test by showing enrichment or depletion of gene expression relative to the "whole hippocampus" fraction. Therefore, our RT-qPCR reveals a purified astrocytes isolation from hippocampus of adult rats by showing enriched expression of GFAP compared to the hippocampus fraction (all cells) with depletion expression of ITGAM, RBFOX3 and MBP genes at all-time points.

(a) Purified astrocyte isolation from the hippocampus of adult rats at four hours post single blast wave exposure by showing enriched expression of GFAP compared to hippocampal fraction sample with depletion expression of ITGAM, RBFOX3, and MBP genes.

(b) Purified astrocyte isolation from the hippocampus of adult rats at 24 hours post single blast wave exposure by showing enriched expression of GFAP compared to hippocampal fraction sample with depletion expression of ITGAM, RBFOX3, and MBP genes.

(c) Purified astrocyte isolation from the hippocampus of adult rats at three days post single blast wave exposure by showing enriched expression of GFAP compared to hippocampal fraction sample with depletion expression of ITGAM, RBFOX3, and MBP genes.

(d) Purified astrocyte isolation from the hippocampus of adult rats at seven days post single blast wave exposure by showing enriched expression of GFAP compared to hippocampal fraction sample with depletion expression of ITGAM, RBFOX3, and MBP genes.

(n=12/marker; data is represented as normalized by "whole hippocampus fraction";)

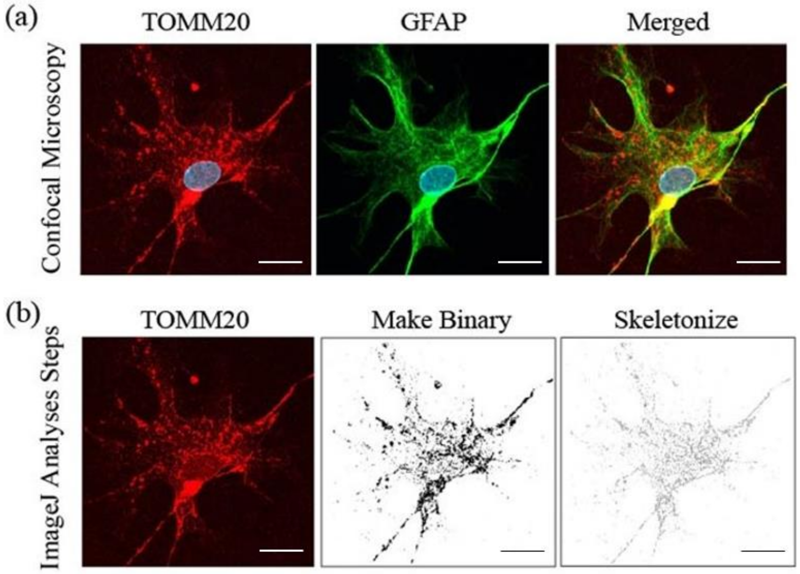

Supplementary Figure S3: Mitochondrial Network Analysis (MiNA) software description and image acquisition.
(a) Representative images from the 4-hour time point 2D primary astrocytes stained for TOMM20 (red) marking an outer mitochondria membrane protein, GFAP (green) a marker for astrocytes and DAPI (blue) to determine the number of nuclei. All pictures were acquired by a 63x/1.40 oil objective. Z- stack series considered of 0.25 µm slice intervals with 12 slices per sample. Images were stacked into a single 2D image using the Zeiss Zen blue 2 software processing tool called "extended depth of focus." Mitochondrial morphological parameters were further measured using Fiji as previously (Image J) disclosed **(62)**. The mitochondria morphology is classified as individuals or networks. Individuals consist of structures without a junction pixel, while networks contain branches with one or more junction pixels.
(b) This figure shows how a 2D picture of primary astrocytes was processed such that mitochondrial network features could be obtained. The original image is processed on ImageJ and then binarized and skeletonized for data acquisition. The same steps are conducted for all time points and for the sham and blast groups (Scale bar = 10 µm).

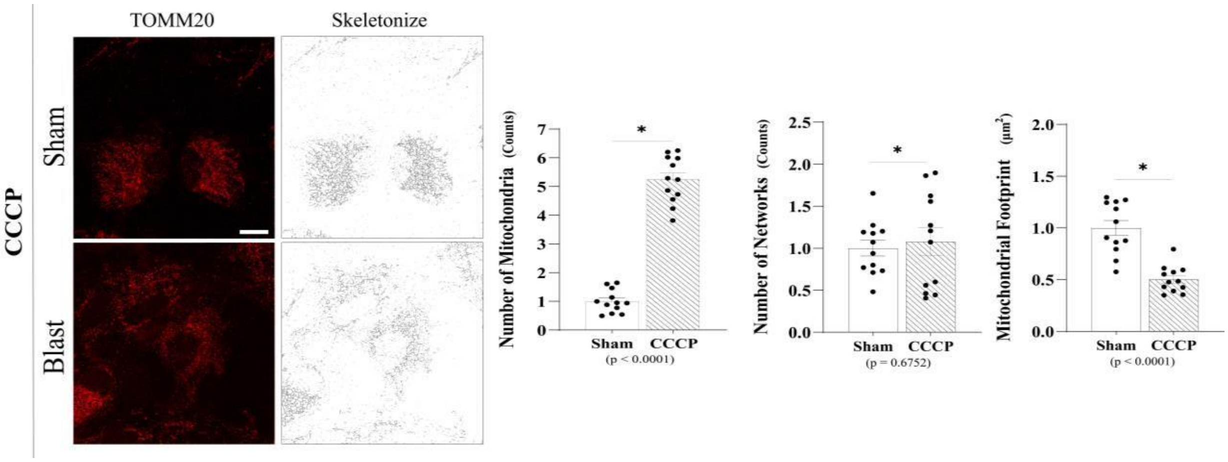

Supplementary Figure S4: Mitochondrial Network Analysis (MiNA) software descriptors show differences in astrocyte mitochondrial morphology in Sham and CCCP positive control groups.
Representative images of TOMM20 and skeletonized for data acquisition. Astrocytic mitochondria treated with 10 µM CCCP (positive control) were severely fragmented with significantly reduced mitochondrial fragment length and total mitochondrial footprint. It further shows that MiNA successfully identified and characterized morphological features of mitochondrial networks.
(Each point represents average of 3 cells per image with a total of 4 images per group; data are mean ±SEM; *p-value represents ≤ 0.05; Scale bar = 10 µm).

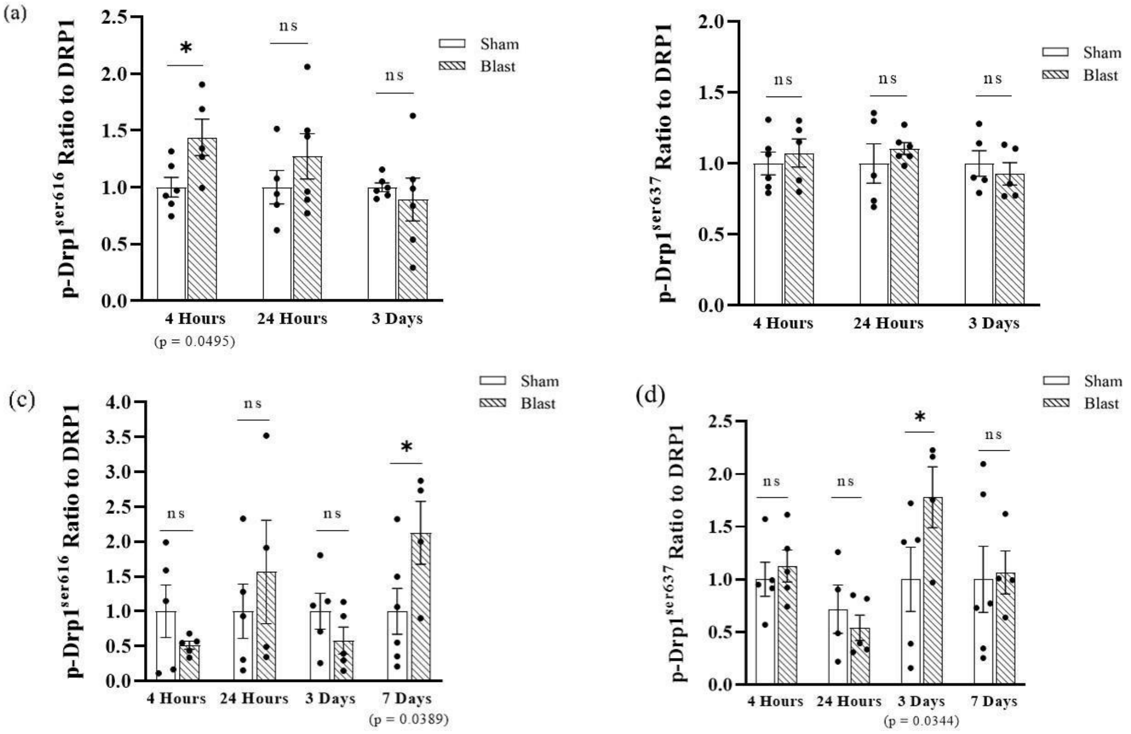

Supplementary Figure S5: Analysis of p-DRP1^{s616} and p-DRP1^{s637} protein ratio to total DRP1 post single mechanical exposure. Western Blotting was conducted using an automatic capillary-based system (Wes, Protein Simple) to look at relative protein quantification to identify possible fission post-overpressure exposure.

(a) ***In Vitro*****:** The ratio of p-DRP1s^{616} protein were significantly increased by hour hours post single mechanical exposure.

(b) ***In Vitro*****:** The ratio of p-DRP1^{s637} protein were maintained to physiological levels at all-time points post single mechanical exposure.

(c) ***In Vivo*****:** The ratio of p-DRP1s^{616} protein were significantly increased by seven days post single mild blast wave exposure.

(d) ***In Vivo*****:** The ratio of p-DRP1^{s637} protein were significantly increased by three days post single mechanical exposure.

(n = 6/group; data are mean ± SEM; *p-value represents ≤ 0.05; ns-value represents not significant).

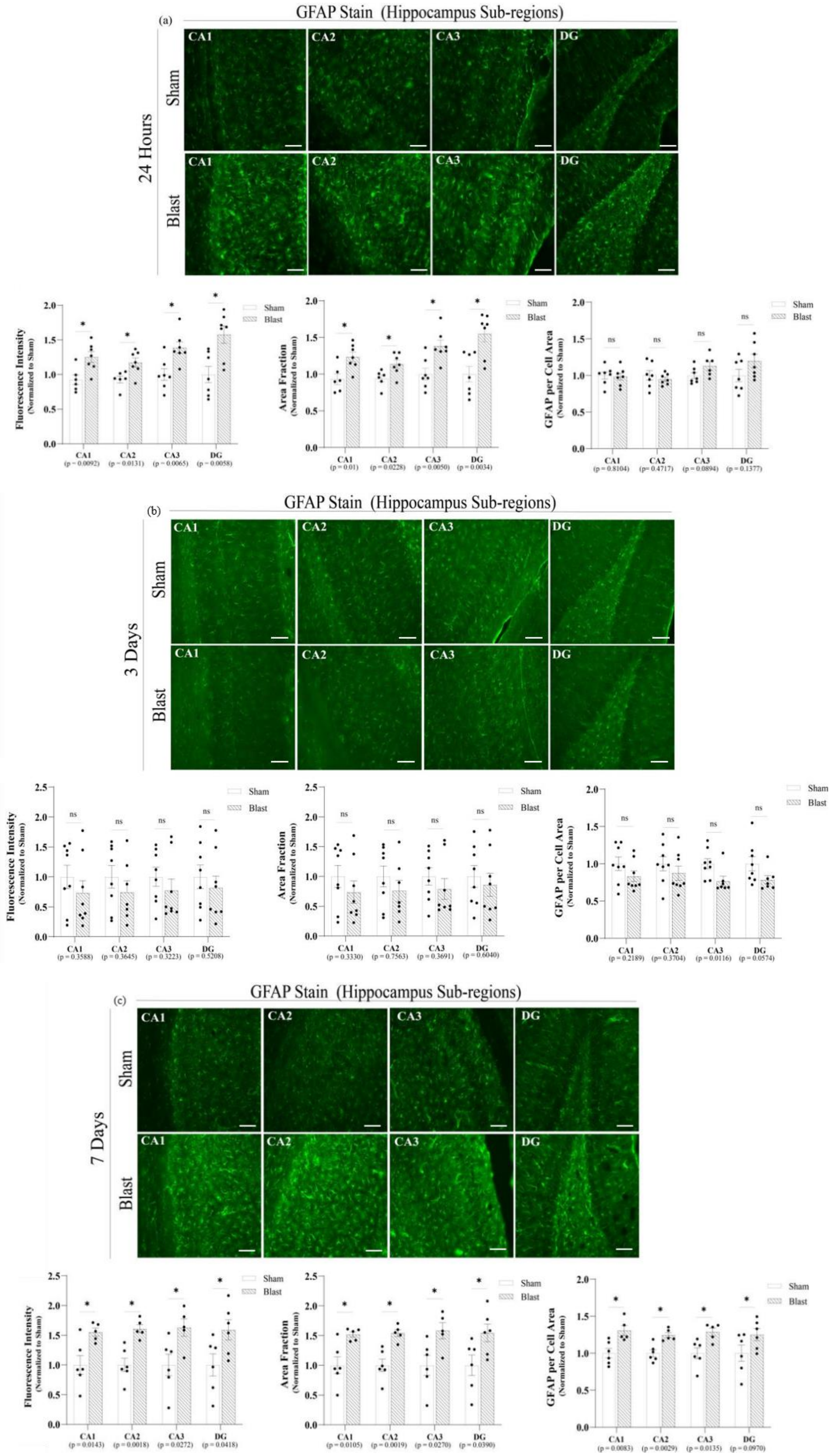

Supplementary Figure S6: Sub-regions of the hippocampus were further analyzed for GFAP to identify if the total hippocampus data correctly represent a diffuse injury across the adult rodent hippocampus at 24 hours, three days and seven days post single mild blast wave exposure. Image was collected from each hippocampal sub-region: Dentate gyrus (DG) and Cornu Ammonis 1, 2, and 3 (CA1, CA2, and CA3). Three parameters were used by

ImageJ Software in order to quantify astrocyte reactivity (Integrated density of fluorescence, area fraction and mean are per cell.

(a) Representative images of GFAP hippocampal sub-region obtained from 10 weeks old adult male rats. At 24 hours, blast presented a higher fluorescence intensity across all hippocampal sub-regions compared to sham group, indicating an initial acute reactive phenotype.

(b) Representative images of GFAP hippocampal sub-region obtained from 10 weeks old adult male rats. At 3 days, blast group presented no significant changes compared to sham across all hippocampus sub-regions.

(c) Representative images of GFAP hippocampal sub-region obtained from 10 weeks old adult male rats. At 7 days, blast group presented an increase within all quantified parameters across all hippocampal sub-regions compared to sham. This Indicated a shift back to a reactive phenotype at the sub-acute stages of the injury.

(Fluorescence Microscopy group: n=8/group; data are mean ± SEM; *p-value represents ≤ 0.05; ns-value represents not significant; Scale bar in a = 57µm).
